# Intracellular delivery of fluorescent protein into viable wheat microspores using cationic peptides

**DOI:** 10.3389/fpls.2015.00666

**Published:** 2015-08-28

**Authors:** Andriy Bilichak, Justin Luu, François Eudes

**Affiliations:** Lethbridge Research Centre, Agriculture and Agri-Food CanadaLethbridge, AB, Canada

**Keywords:** microspores, cell-penetrating peptides, R9, direct protein delivery, wheat

## Abstract

Microspores are specialized generative cells with haploid genome that demonstrate the amenability toward embryogenesis under certain conditions. The induced microspore culture technique is largely exploited by the breeding programs of wheat and other crops due to its high efficiency for generation of the large number of haploid plants in the relatively short period of time. The ability to produce mature double haploid plant from a single cell has also attracted attention of the plant biotechnologists in the past few years. More importantly, the possibility to deliver proteins for improvement of embryogenesis and the genome modification purposes holds great potential for transgene-free wheat biotechnology. In the present study, we examined the ability of cationic and amphipathic cell penetrating peptides (CPPs) to convey a covalently-linked mCherry protein inside the viable microspores. We demonstrate that the affinity of CPPs to the microspore cells dependents on their charge with the highest efficiency of CPP-mCherry binding to the cells achieved by cationic CPPs (penetratin and R9). Additionally, due to overall negative charge of the microspore cell wall, the successful uptake of the protein cargo by live microspore cells is attained by utilization of a reversible disulfide bond between the R9 CPP and mCherry protein. Overall, the approach proposed herein can be applied by the other biotechnology groups for the fast and efficient screening of the different CPP candidates for their ability to deliver proteins inside the viable plant cells.

## Introduction

Bread wheat (*Triticum aestivum* L.) is one of the major economically important crops worldwide that provides around one fifth of the calories to human population (Wang et al., 2014a). The creation of necessary genetic diversity, introduction of the new traits and the generation of improved cultivars for wheat involves predominantly classical breading. The last, in turn, relies on the techniques which can generate a large number of haploid plants either by pollination of wheat with alien species (*Hordeum bulbosum*, maize or sorghum) (Barclay, 1975; Laurie and Bennett, 1988a,b) or by microspore and anther culture. Despite some benefits of the wheat pollination technique, the microspore culture produces the large number of haploid plants in the shorter period of time (Touraev et al., 1996; Hul and Kasha, 1997; Hu and Kasha, 1999). More importantly, being a synchronized population of single cells with haploid genome and morphogenic potential, the microspore technique offers enormous prospective for improvement of embryogenesis in the culture as well as the possibility of genome editing at a single-cell level. Substantial effort has been made to develop methods for transformation of microspores and microspore-derived tissue. Overall, depending on the physiological stage that transformation occurs at, the procedures can be classified as: gametophytic and sporophytic (Resch and Touraev, 2010). Whereas the former one encompasses pollination using either the transformed mature pollen or *in vitro* cultured pollen grains derived from transformed microspores, the latter one utilizes haploid microspore-derived embryos as the plant material for stable transformation. In most of the cases the transformation is performed using either *A. tumefaciens* or electroporation or bombardment (Brisibe et al., 2000; Folling and Olesen, 2001; Kumlehn et al., 2006; Chauhan and Khurana, 2011; Brew-Appiah et al., 2013). While being efficient for the introduction of foreign DNA in the wheat genome, unfortunately, neither of these techniques currently offers the possibility of protein delivery into viable microspore cells. Direct introduction of the protein molecules into microspores at the certain physiological stage can potentially be utilized for improvement of the culture embryogenesis and generation of the higher number of green haploid plants for the breeding programs. The broader application includes the exploitation of the designed endonucleases (i.e., Zinc Finger Nucleases, Transcription activator-like effector nucleases, CRISPR/Cas9, and meganucleases) in the form of proteins for the permanent introduction of mutations at the selected loci. Moreover, delivery of the functional enzymes in the form of proteins eliminates the possibility of generating the plants containing transgenes and the resulting product is expected to be void of regulation covering GMO crops (Voytas and Gao, 2014).

An alternative technique for the cargo delivery into live cells has been proposed in the recent years that utilizes the natural ability of cell penetrating peptides (CPPs) to internalize protein, DNA or nanoparticles into animal and plant cells (Chang et al., 2005, 2014; Liu et al., 2013). The first CPP, discovered in 1988, was the domain of transcriptional activator protein (Tat) of the human immunodeficiency virus type 1 (HIV-1) (Frankel and Pabo, 1988; Green and Loewenstein, 1988). Later on, it has been established that the presence of the basic amino acids in the CPP sequence is prerequisite for its translocation through the plasma membrane and accumulation in the cytoplasm and nucleus (Vivès et al., 1997). Subsequently, a number of naturally-occurring and synthetic CPPs were identified with the different properties capable of delivering a wide range of bioactive molecules into the living cells (Fonseca et al., 2009; Huang et al., 2013; Chang et al., 2014; Wang et al., 2014b). Depending on the origin they can be classified as protein-derived, synthetic and chimeric (Lindgren and Langel, 2011). Additionally, based on their physical-chemical properties CPPs can be divided into amphipathic, hydrophobic, and cationic peptides.

The interaction between CPP and the cargos can be either covalent or non-covalent. Whereas, the former one is usually made through disulphide bridges, ester or peptide bonds, the latter one relies on the weak van der Waals forces, electrostatic interactions, and hydrophobic effects (Chang et al., 2014). Previously, we have shown that cationic peptides (Tat and Tat2) are able to deliver protein and DNA molecules in the form of non-covalent complexes into triticale microspores (Chugh et al., 2009). Nevertheless, it still remains vague whether the covalently-linked CPPs can convey the protein molecules in their native state inside the live microspores and how CPPs with different physical-chemical properties interact with the microspore cells. In the present study, we selected two cationic [penetratin (Derossi et al., 1994) and R9 (Futaki, 2002)] and two amphipathic [transportan (Pooga et al., 1998) and model amphipathic peptide (Oehlke et al., 1998)] CPPs and examined their ability to deliver functional covalently-linked mCherry protein into live microspore cells. For this, in-frame fusion of proteins with CPP residues at the N-terminus were purified from bacterial culture and the screening for the best peptide for mCherry conveyance inside the microspore cells was conducted. The data presented in this communication reveals the drastic difference between CPPs for their ability to interact with microspores as well as the importance of utilization of the temporary covalent bonds for the protein cargo internalization inside viable cells.

## Materials and methods

### Plant cultivation

Wheat seeds (cultivar AC Andrew) were germinated and grown in the Cornell mix as described in Asif et al. (2013) and Sinha and Eudes (2015). The plants were treated with 2.5 ml/l of Tilt™ (propiconazole, Syngenta) before the tillering stage No. 2 as per (Zadoks et al., 1974) and Intercept™ (0.004 g/l of soil, Imidacloprid, Bayer) once sufficient root development was established to control pests. The tillers were harvested when the microspores reached mid-to-late uninucleate stage. The tillers were kept at 4°C for 3 weeks with their bases in distiled water and their heads wrapped in aluminum foil. Following 3 weeks ± 3 days of pre-treatment, the spikes were extracted from their tillers and after evaluation of their general appearance, only the most homogenous spikes were used for microspores isolation.

### Microspores isolation

Isolation of microspores was done as described previously (Asif et al., 2013; Sinha and Eudes, 2015). Briefly, the stage of microspores was verified using a median floret and an acetocarmine staining. Twelve spikes per extraction were surface sterilized with 10% (v/v) bleach (5.25% sodium hypochlorite) for 3 min followed by three washes with sterile double distiled water with constant agitation. Florets were aseptically dissected and transferred to a sterile and refrigerated 110 ml Warring blender cup (VWR International) containing 50 ml of filter sterilized extraction solution at 4°C (14 mM KNO_3_, 1.76 mM (NH_4_)_2_SO_4_, 1.47 mM KH_2_PO_4_, 0.56 mM CaCl_2_, 0.38 mM MgSO_4_, 0.1 mM FeSO_4_, 0.1 mM Na_2_EDTA, 0.9 mM MES and 400 mM mannitol, pH 6.5). The florets were blended twice for 7 s at low speed (18,000 rpm). The extract was filtered through 100 μm sterile mesh (VWR International) and the cells were then pelleted by centrifugation (100 g for 5 min at 4°C) using a swinging bucket rotor (Eppendorf AG, Hamburg, Germany). After removing the supernatant, the pellet was resuspended in 16 ml of ice-cold extraction solution. Following a second spin (100 g for 5 min at 4°C), the microspore cells were resuspended in 6 ml of pre-chilled, sterile 20% maltose solution and 1 ml of extraction medium was layered on top. The content was centrifuged at 100 g for 13 min at 4°C and the viable microspore cells in the interphase were transferred to the fresh 15 ml tube. The microspores were washed again with 15 ml of extraction solution and resuspended to the final concentration of 500 cells per microliter.

### Plasmid constructions

Protein expression plasmids pET45b(+)::Cys-mCherry and pET45(+)::CPP-Cys-mCherry were generated by 5′ in-frame fusion of sequences coding either for cysteine (Cys) or CPPs (either R9, penetratin, transportan or model amphipathic peptide) followed by Cys to mCherry coding sequence using PCR (mCherry GenBank ID: JN717246.1). Since mCherry protein does not contain cysteinyl residues, the codon for cysteine was introduced into CDS in order to facilitate reversible covalent linkage formation with thiol-reactive nitropyridyl (Npys) Arg_9_ peptide (Cys (Npys)-(D-Arg)_9_). A 3′-primer contained a sequence coding for six histidines to aid in the downstream protein purification (Supplementary Table 1). The Cys-mCherry and CPP-Cys-mCherry PCR products were cloned as *Nco*I and *Hind*III fragments into linearized pET45b(+) plasmid. The recombinant constructs were verified by sequencing.

### Protein overexpression and purification from bacterial culture

The constructed plasmids were transformed into *E.coli* strain BL21 (DE3) (Novagen, USA). Single colony of every construct was inoculated into 25 ml of LB (the starter culture) and was grown overnight at 37°C with shaking at 250 rpm in the presence of 100 μg/ml of carbenicillin. The following day, the starter culture was transferred to 500 ml of LB supplemented with carbenicillin and was grown at 37°C until OD_600_ reached 0.5. The culture was moved to 16°C and the protein expression was induced with 1 mM IPTG. Following 18 h of protein induction, the bacterial culture was harvested by centrifugation and the pellet was re-suspended in 25 ml of a His-Tag buffer A (20 mM HEPES, 0.3 M NaCl, 5% glycerol and 40 mM imidazole, pH 7.5) supplemented with EDTA-free protease inhibitor cocktail (Roche). Cells were disrupted by sonication (50% intensity, 30 s on, 1 min off time, four cycles, model Q55, Qsonica), and the insoluble debris were removed by centrifugation (18,500 g, 1 h at 4°C). The clarified supernatant was applied directly onto 1 ml His-Trap column (GE Healthcare Life Sciences) equilibrated with the His-Tag buffer A using an FPLC system (AKTA purifier, GE Healthcare Life Sciences). The elution of bound protein was done using a linear gradient of His-Tag buffer B (20 mM HEPES, 0.3 M NaCl, 5% glycerol and 500 mM imidazole, pH 7.5) from 0 to 100%. Fractions containing the target protein (assessed by the mCherry absorbance at 587 nm) were combined together and concentrated till 0.5 ml using 10K Amicon Ultra-15 spin concentrators (EMD Millipore). The protein was filtered through 0.2 μm low-protein binding filter (EMD Millipore) and loaded onto HiLoad 16/60, Superdex 200 prep grade size exclusion column (SEC, GE Healthcare Life Sciences) primed with SEC buffer (20 mM HEPES, 0.15 M NaCl, pH 7.5) using FPLC system. Following gel filtration step the fractions containing CPP-mCherry protein were analyzed on 10% SDS-PAGE gel. The fractions with more than 90% purity were combined together and concentrated using spin concentrators till at least 50 μM. Glycerol was added to the fractions till the final concentration of 5% and the proteins were stored at −80°C.

### R9-mCherry complex formation

To prepare R9-mCherry complex with the covalent bond through disulfide bridge, purified Cys-mCherry protein (50 μl; 1 nmol in HEPES SEC buffer, pH 7.5) and up to 30 nmol of Cys-(Npys)-(D-Arg)_9_ peptide in 50 μl of water (AnaSpec) were combined and allowed to react at room temperature for at least 1 h with no mixing (Liu et al., 2014). The non-covalent complex formation was done by mixing Cys-mCherry protein (50 μl; 1 nmol in HEPES SEC buffer, pH 7.5) and 1 nmol of L-Arg_9_ peptide in 50 μl of water (AnaSpec). The solution was incubated for 1 h with no mixing at room temperature.

### Microspores treatments

Microspores were treated either with in-frame fusion or *in vitro* combined Arg_9_-mCherry protein. Protein solutions (up to 2 nmol in 100 μl) were mixed with 100 μl of microspores (50,000 cells) in extraction buffer and incubated for 1 h (unless otherwise indicated) at room temperature. Following incubation, microspores were washed three times with 2 ml each of extraction buffer to remove the excess of protein. Microspores were resuspended in 200 μl of extraction buffer and fluorescein diacetate (FDA) was added till 2.4 μM in order to visualize viable cells (Sinha and Eudes, 2015). All experiments were done in three biological replicates.

### Collecting the data using plate reader and microscopy imaging

Microspores viability and the level of intensity after treatment with CPP-Cys-mCherry proteins were measured using BioTek Synergy Mx microplate reader (BioTek Instruments, Inc., USA). For this, the microspore solution was resuspended and 12,500 cells in 50 μl volume were pipetted into every well of the black 96-well plate (Costar, Fisher Scientific). For FDA and mCherry measurements, the fluorescent reading was done at excitation/emission wavelengths of 492/517 and 587/610 nm, respectively. The data normalization was done by setting the fluorescent reading from the FDA measurement in the untreated control sample to 100%. For the mCherry signal detection, the reading from untreated control was set to 1 and the fold difference as compared to control was calculated. No cross-reading was detected between two fluorescent signals under aforementioned conditions. All measurements were done in three biological and two technical repeats.

The visual examination of treated microspores was done using both EVOS FL cell imaging system (Life Technologies) and confocal laser scanning microscope Olympus, FV1000 (Olympus).

### Statistical analysis

The statistical significance was confirmed by single-tailed paired Student's *t*-test with α = 0.05 and the single factor ANOVA.

## Results

### Selection of CPPs for in-frame fusion with mCherry protein

**Figure 1:**
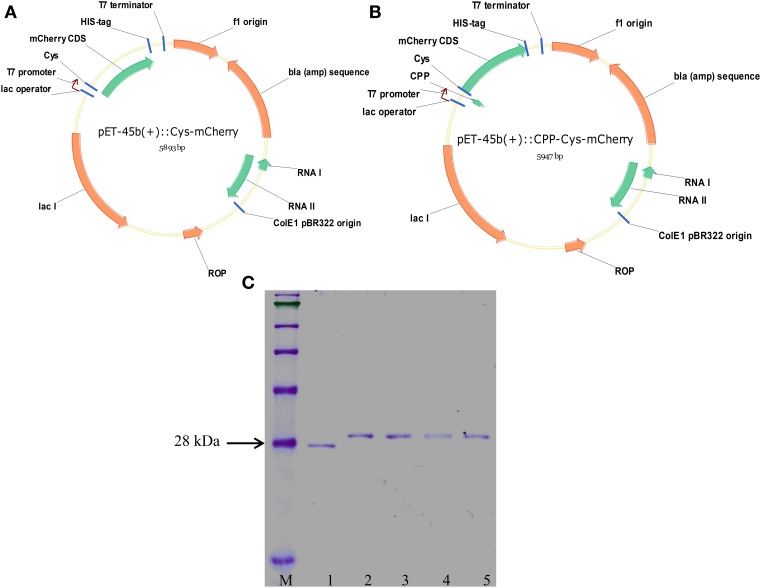
**Schematic map of generated vectors used for purification of Cys-mCherry (A) and CPP-Cys-mCherry (B) proteins from bacterial culture**. **(C)** SDS-PAGE analysis of the purified proteins: 1, Cys-mCherry; 2, penetratin-Cys-mCherry; 3, R9-Cys-mCherry; 4, transportan-Cys-mCherry; 5, MAP-Cys-mCherry; M, molecular weight protein marker.

The mCherry protein was chosen as a cargo due to its stability, fast maturation time, high level of fluorescence, and low auto fluorescence level of microspores in the mCherry spectrum range. Four distinct CPPs were picked up for microspore transfection experiments based on their physical-chemical properties (Table 1). The nucleotide sequences coding for corresponding CPPs and 6xHis sequence were fused in-frame to the coding sequence of mCherry at the 5′- and 3′-ends, respectively, using PCR. The resulting PCR fragments were cloned into pET45b(+) plasmid (Figures 1A,B). The proteins were purified from bacterial culture with the final purity of more than 98% (Figure 1C). All mCherry fusions demonstrated same intensity of fluorescence in regard to their concentration indicating that CPPs did not affect the protein confirmation (Supplementary Figure 1). The mCherry protein bearing the cysteine at the N-terminus was used as a no-CPP control treatment for all microspore transfection experiments.

**Table 1:** **Characteristics of CPP-mCherry proteins used in the current study**.

**Name of the fusion protein**	**CPP sequence**	**CPP origin**	**Isoelectric point of the protein**	**Charge of the protein at pH7**	**Molecular weight of the protein, kDa**	**Percentage of hydrophobic amino acids by weight in the protein**	**References**
Cys-mCherry	–	–	6.1	−5.2	27.9	25.4	
Penetratin-Cys-mCherry	RQIKIWFQNRRMKWKK	Protein-derived	7.9	1.8	30.1	25.9	Derossi et al., 1994
R9-Cys-mCherry	RRRRRRRRR	Synthetic	8.7	3.8	29.3	24.2	Futaki, 2002
Transportan-Cys-mCherry	GWTLNSAGYLLGKINLKALAALAKKIL	Semi-synthetic	6.7	−1.16	30.7	28.1	Pooga et al., 1998
MAP-Cys-mCherry	KLALKLALKALKAALKLA	Synthetic	7.0	−0.16	29.8	28.0	Oehlke et al., 1998

### The interaction of CPP-mCherry proteins with microspore cells

**Figure 2:**
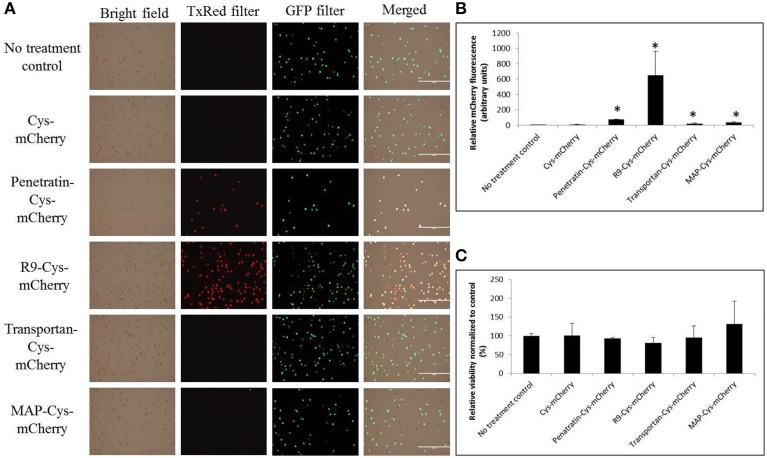
**The effect of different CPPs on the binding capacity of the CPP-Cys-mCherry proteins to the microspore cells. (A)** Microscopy imaging of the treated microspores. Microspores were subjected to incubation with 1 nmol of one of the proteins for 1 h, followed by three washing steps and examination using EVOS FL cell imaging system (Life Technologies). The TxRed and GFP LED light cubes were used to visualize fluorescent signals from mCherry and FDA stain, respectively. The horizontal bars on the merged images indicate 1 mm scale. **(B,C)** Quantification of intensity of fluorescent signals for mCherry and FDA chromophores, respectively, using BioTek Synergy Mx microplate reader. Data are shown as average calculated from three independent repeats with SD. For the data normalization see the Materials and Methods Section. The stars indicate statistically significant difference as compared to Cys-mCherry treatment (Student's *t*-test, α = 0.05).

Two cationic (R9 and penetratin) and two amphipathic (transportan and MAP) peptides were used to test their ability to translocate covalently linked mCherry protein into viable microspore cells. Treatment of microspores with equal amounts (1 nmol each) of either Cys-mCherry or CPP-Cys-mCherry proteins for 1 h, followed by examination using fluorescent microscopy, revealed that both cationic CPPs demonstrate the highest binding to the negatively charged microspore's exine as compared to amphipathic ones (Figure 2A). The amphipathic CPP-Cys-mCherry proteins showed interaction with the microspore cell wall that was the lowest among CPP-mCherry fusions tested. Treatment of the cells with the R9-Cys-mCherry protein displayed the highest intensity of fluorescent signal among tested CPP-fusion proteins (Figure 2B, Student's *t*-test, *P* < 0.05), hence the R9-fusion protein was selected as the candidate for the further detailed examination of its CPP properties. No decrease in microspore viability was observed for any of the treatments under tested conditions (Figure 2C, Student's *t*-test, *P* > 0.05).

### Evaluation of the R9-Cys-mCherry interaction with the wheat microspores

**Figure 3:**
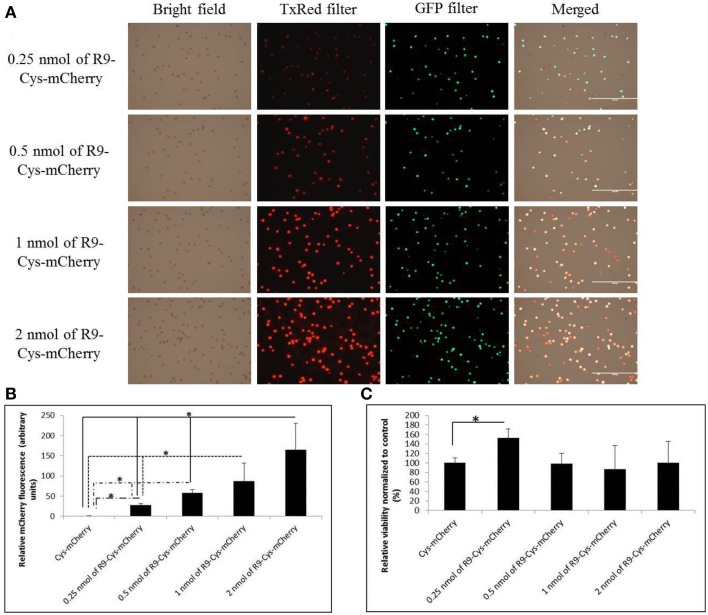
**Evaluation of the binding capacity of the R9-Cys-mCherry protein to the microspore cells**. **(A)** Microscopy imaging of the microspores treated with different amounts of the R9-Cys-mCherry protein. **(B,C)** Quantification of the fluorescence intensity from the mCherry and FDA chromophores, respectively, using BioTek Synergy Mx microplate reader. Data are shown as average calculated from three independent repeats with SD. For the data normalization see the Materials and Methods Section. The stars indicate statistically significant difference between connected bars (Student's *t*-test, single factor ANOVA, α = 0.05; *R*^2^ = 0.86 for treatments displayed in **B**).

**Figure 4:**
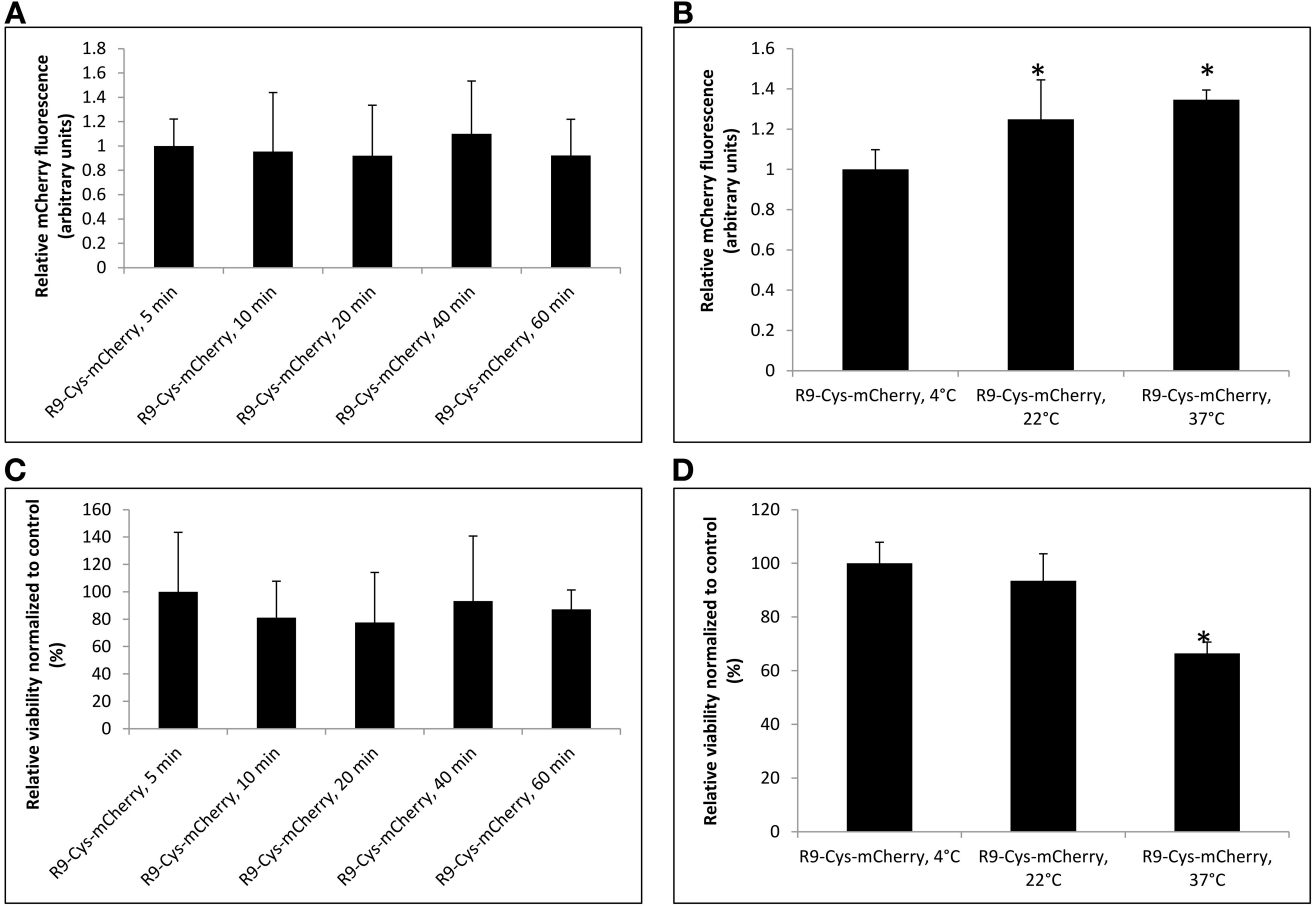
**The effect of different time and temperature treatments on the binding capacity of the R9-Cys-mCherry protein to the microspore cells (A,B, respectively) and on the microspore viability (C,D)**. For all experiments the treatment was done with 1 nmol of the R9-Cys-mCherry protein, followed by microspore washing for three times and quantification using BioTek Synergy Mx microplate reader. Data are shown as average calculated from three independent repeats with SD. For the data normalization see the Materials and Methods Section. The stars indicate statistically significant difference as compared to untreated control (Student's *t*-test, α = 0.05).

Further analysis of the R9-Cys-mCherry binding to the microspore cells demonstrated that the effect was dose-dependent (Figure 3A) and the intensity of fluorescent signal demonstrated linear correlation with the amount of applied protein in a range from 0.25 to 2 nmol (Figure 3B, *R*^2^ = 0.86). The viability of the treated microspores was not compromised under the treatment conditions (Figure 3C). The interaction of R9-Cys-mCherry with the cells apparently occurred mostly on the surface of the cells, since as little as 5 min was required for the fluorescent signal to achieve its maximum intensity when 1 nmol of the protein was used (Figure 4A, Student's *t*-test, *P* > 0.1). Furthermore, the binding of the protein to the microspore outer surface was temperature-dependent (Figure 4B), although the microspore viability was significantly compromised when the treatment was performed at 37°C for 1 h (Figure 4D).

### Temporary covalent bond between R9 and mCherry is required for protein cargo delivery into viable wheat microspores

**Figure 5:**
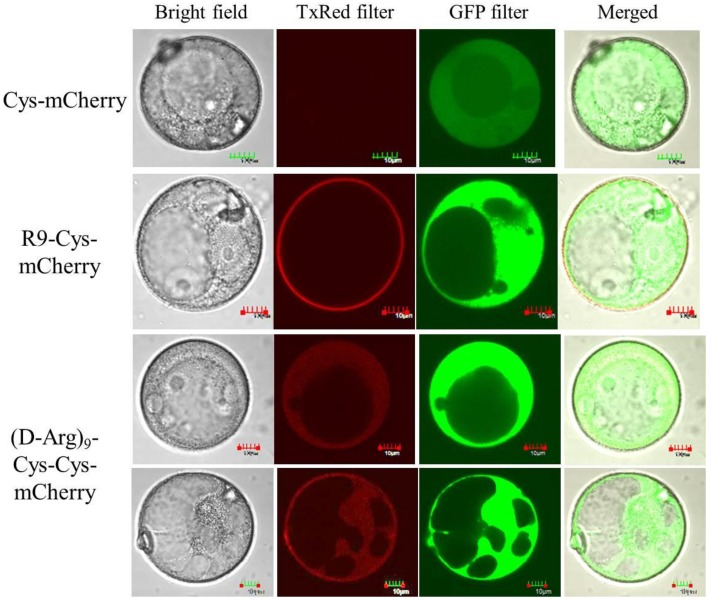
**Analysis of mCherry localization in the microspore cells using confocal laser scanning microscopy**. Microspores were incubated with either Cys-mCherry, R9-Cys-mCherry or (D-Arg)_9_-Cys-Cys-mCherry complex at 1:1 molar ratio overnight at room temperature. Following incubation, the cells were washed and stained with 2.4 μM FDA to visualize viable microspores. The TxRed and GFP filter sets were used to capture the fluorescent signals from mCherry and FDA, respectively.

**Figure 6:**
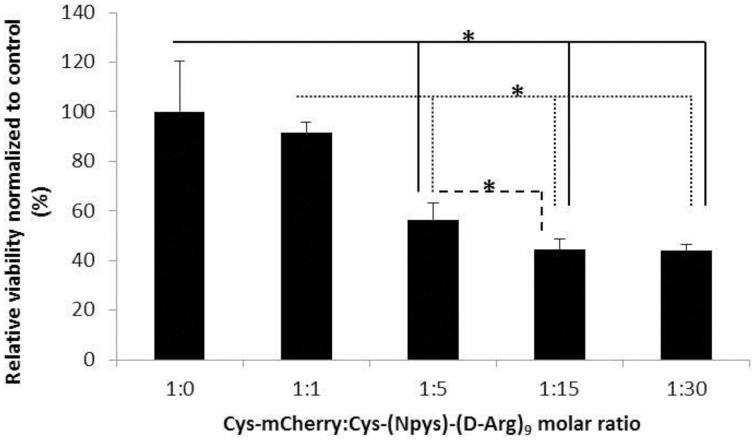
**Evaluation of Cys (Npys)-(D-Arg)_9_ cytotoxicity on the microspore cells**. The Cys-mCherry—CPP complex formation was done for 1 h in 100 μl volume by combining 1 nmol of the protein with the different amounts of Cys (Npys)-(D-Arg)_9_ in indicated molar ratios. Later, 50,000 cells in 100 μl volume were added to the reaction and the solution was incubated for 1 h at room temperature. Following the washing steps, the viability of microspores was quantified using FDA assay and the microplate reader. The data normalization was done by setting the fluorescent reading from the sample treated with the Cys-mCherry alone to 100%. Data are shown as average calculated from three independent repeats with SD. The stars indicate statistically significant difference between connected bars (Student's *t*-test, single factor ANOVA, α = 0.05, *R*^2^ = 0.64).

Detailed examination of the microspores treated with R9-Cys-mCherry using confocal laser scanning microscopy revealed that the majority of the protein was trapped on the exine of microspores (Figure 5). We hypothesized that the negative charge of exine (Salter et al., 2002) caused a strong binding of the R9-Cys-mCherry protein to the surface of the cells and supplementing of R9 peptide that can form a reversible covalent bond with the target protein is required for the protein to be internalized inside the cells. Having an additional cysteinyl residue at the N-terminus of the protein allowed us to attach an R9 peptide [Cys (Npys)-(D-Arg)_9_] through asymmetrical disulfide bond that can be dissociated under reducing conditions inside a cell (Liu et al., 2014). The unreacted CPPs may, apparently, coat Cys-mCherry molecules through electrostatic interaction and, thus, further aid in the translocation of the protein through exine and plasma membrane by making the mCherry protein overall positively charged. Due to the known cytotoxicity of CPPs (Scheller et al., 1999), we first examined the effect of Cys (Npys)-(D-Arg)_9_ peptide on the microspore viability. The conjugation reaction was performed for at least 1 h at room temperature as described previously (Liu et al., 2014). The microspore cells demonstrated elevated sensitivity to the tested peptide at the Cys-mCherry to CPP molar ratios higher than 1:1 (Figure 6). Hence, the molar ratio of 1:1 was used for the transfection of microspores [1 nmol of Cys-mCherry protein to 1 nmol of Cys (Npys)-(D-Arg)_9_ peptide]. Consistent with our hypothesis, supplementing of D-R9 peptide which was able to form disulfide bridges with the mCherry protein resulted in detection of viable microspores with internalized Cys-mCherry protein as revealed by confocal laser scanning fluorescent microscopy (Figure 5). The Cys-mCherry protein was predominantly localized to the cytoplasm and nucleus of the cells and was excluded from the vacuoles. In contrast, we were not able to detect transfected microspores when non-covalent complex formation was performed using L-R_9_ peptide (1 nmol of Cys-mCherry protein to 1 nmol of L-Arg_9_ peptide). No fluorescent signal was detected when microspores were treated with Cys (Npys)-(D-Arg)_9_ peptide alone (data not shown).

## Discussion

The ability to deliver proteins into embryogenic cells/tissues holds a great potential both for academic and applied studies. Due to the relatively short half-life of proteins, the necessary effects can be achieved transiently and at a particular stage of the cell development. The possible implications include temporary changing the fate of the tissue development, enhancing the embryogenesis level, and even permanent modification of the plant genome. The last approach has been successfully established in animal cells through delivery of designed endonucleases in the form of proteins using CPPs for the transgene-free genome editing purpose (Liu et al., 2014; Ramakrishna et al., 2014). In both cases, synthetic R9 peptide has been used for the protein translocation. The net positive charge of the cargo molecule seems to be the requirement for successful cellular uptake in the animal cells. For instance, the relatively large protein molecules like Zinc Finger Nucleases (around 45 kDa) that belong to the family of positive supercharged proteins are able to cross the plasma membrane of the animal cells in active form (Gaj et al., 2012; Liu et al., 2015). At the same time, a number of amphipathic CPPs have been ascribed for the animal cells which are characterized by the helical structure and the presence of lysine residues.

In this study, we made an attempt to compare two different classes of CPPs for their ability to interact with the microspore cells and to internalize protein cargo. Consistent with the previous reports, we observed that cationic peptides demonstrate high affinity for the plant cells (Figures 1A,B) (Chang et al., 2005). For instance, previously, it has been reported that either covalent (Chang et al., 2005) or non-covalent (Chang et al., 2007) complexes with either Tat or R9 were able to convey fluorescent proteins inside the onion and tomato roots and epidermal cells. Additionally, in line with our data neither of the treatments had a cytotoxic effect on the wheat microspores when CPPs where permanently fused to the protein (Figure 1C) (Chang et al., 2005). On the other hand, to our surprise, neither of the amphipathic CPPs demonstrated strong interaction with microspore cells. These data come in the disagreement with the previous report where both cationic (penetratin, Tat_48–60_) and amphipathic (transportan and MAP) CPPs were tested for their ability to internalize covalently linked, through disulfide bond, pentapeptide cargo (labeled with the 2-amino benzoic acid fluorophore) into animal cells (Hallbrink et al., 2001). Both amphipathic CPPs demonstrated the highest level of uptake by Bowes human melanoma cells. It has been hypothesized that the difference in the efficiency of cargo delivery between the amphipathic and cationic CPPs is due to the ability of the former ones to enter and exit cells more easily since they are in equilibrium over the plasma membrane while the members of the latter class are not (Hallbrink et al., 2001). At the same time, the kinetics of CPP trafficking through the membrane is probably dependent not only on peptides, but also on the membrane area and lipid bilayer composition, and consequently is cell-type specific.

The microspore cell is composed of three parts: exine, intine, and protoplast (Tang et al., 2013). The exine of microspores consists of the sporopollenin that is assumed to have phenolics and polyhydroxylated unbranched aliphatics, coupled by ester and ether linkages, which provide this biopolymer with its characteristic resistance to chemical degradation and tolerance to ambient stress conditions (Quilichini et al., 2010). Intine, in turn, is composed of cellulose and pectin. The only access to plasma membrane for the CPP-cargo complex is assumed to be through the single micropore or germ pore (Chugh et al., 2009). The strong binding of cationic CPP-protein fusions like R9-Cys-mCherry and penetratin-Cys-mCherry to the microspore cells is apparently due to high density of negative charges in the sporopollenin matrix that makes it amphiphilic with a strong preference for polar molecules (Salter et al., 2002). This notion is further supported by the positive correlation that we observed between the charge of the proteins used in the current study and the fluorescent signal observed after treatment of microspore cells with CPP-mCherry proteins (*R* = 0.7). Additionally, the binding of R9-Cys-mCherry protein was relatively fast, dose-, and temperature-dependent (Figures 3, 4) that suggests for the solely physical-chemical interaction with the exine of the microspores. This however, possessed a challenge for the protein molecules internalization, since they were predominantly trapped on the outer layer of the cells (Figure 6). We hypothesized that a reversible covalent bond between R9 and mCherry protein would allow us to bring the cargo molecule to the close proximity of the micropore and through the plasma membrane, but the link will be reduced as soon as it enters the cell to accommodate the protein release inside the cytoplasm. Due to cytotoxicity of examined R9 CPP [Cys (Npys)-(D-Arg)_9_] for microspore cells in the amount higher than 1 nmol (Figure 6), the complexes with Cys-mCherry were made at the molar ratios of 1:1. Consistent with our hypothesis, incubation with the (D-Arg)_9_-Cys-Cys-mCherry protein conjugates resulted in the cellular uptake of the fluorescent protein by live microspores (Figure 5). The protein was predominantly localized to cytoplasm and nucleus and was excluded from the vacuoles. At the same time we observed that some cells exhibited marked fluorescence as compared to others. This observation is in agreement with the previous report (Chang et al., 2005) and is apparently due to slightly different physiological stage of development of the cells in population. On the other hand, in contrast to other reports on non-covalent R9-mediated protein delivery into roots, we did not observe a detectable mCherry signal inside viable microspore cells when Cys-mCherry was complexed with L-R_9_ at 1:1 molar ratio (Chang et al., 2007). This was probably due to the fact that we used almost 30 times lower final concentration of L-R9 in our transfection experiments as compared to the published study. The sensitivity of microspores to R9 CPP did not allow us to test higher amounts of this cationic peptide.

Overall, in this communication we have described an approach that can be applied by other labs for examination of CPPs with different properties for their ability to interact with the totipotent cells like microspores. At the same time, we provide evidence that careful investigation of the internalization properties of any CPP-cargo is needed, due to physiological specificity of the specialized plant cells. The utilization of the reversible covalent bonds to convey cargos into the plant cells with the high negative charge of the outer layer is a preferable approach. Additionally, the same methodology can be further exploited for other cationic CPPs which may demonstrate higher internalization activity at the lower cytotoxicity rate. The implication of CPP technology for nano-cargo delivery into plant cells is still in its infancy, but, we believe, it holds a great potential for applied and basic studies due to its simplicity, high efficiency and an ability to deliver a wide spectrum of bioactive molecules into plant cells.

### Conflict of interest statement

The authors declare that the research was conducted in the absence of any commercial or financial relationships that could be construed as a potential conflict of interest.

## References

[B1] Asif M. Eudes F. Goyal A. Amundsen E. Randhawa H. Spaner D. (2013). Organelle antioxidants improve microspore embryogenesis in wheat and triticale. In Vitro Cell. Dev. Biol. Plant 49, 489–497. 10.1007/s11627-013-9514-z 23896731

[B2] Barclay I. R. (1975). High frequencies of haploid production in wheat (*Triticum aestivum*) by chromosome elimination. Nature 256, 410–411. 10.1038/256410a0

[B3] Brew-Appiah R. A. Ankrah N. Liu W. Konzak C. F. von Wettstein D. Rustgi S. (2013). Generation of doubled haploid transgenic wheat lines by microspore transformation. PLoS ONE 8:e80155. 10.1371/journal.pone.0080155 24260351 PMC3832437

[B4] Brisibe E. A. Gajdosova A. Olesen A. Andersen S. B. (2000). Cytodifferentiation and transformation of embryogenic callus lines derived from anther culture of wheat. J. Exp. Bot. 51, 187–196. 10.1093/jexbot/51.343.187 10938825

[B5] Chang M. Chou J. C. Lee H. J. (2005). Cellular internalization of fluorescent proteins via arginine-rich intracellular delivery peptide in plant cells. Plant Cell Physiol. 46, 482–488. 10.1093/pcp/pci046 15695452

[B6] Chang M. Chou J. C. Chen C. P. Liu B. R. Lee H. J. (2007). Noncovalent protein transduction in plant cells by macropinocytosis. New Phytol. 174, 46–56. 10.1111/j.1469-8137.2007.01977.x 17335496

[B7] Chang M. Huang Y. W. Aronstam R. S. Lee H. J. (2014). Cellular delivery of noncovalently-associated macromolecules by cell-penetrating peptides. Curr. Pharm. Biotechnol. 15, 267–275. 10.2174/1389201015666140617095415 24938892

[B8] Chauhan H. Khurana P. (2011). Use of doubled haploid technology for development of stable drought tolerant bread wheat (*Triticum aestivum* L.) transgenics. Plant Biotechnol. J. 9, 408–417. 10.1111/j.1467-7652.2010.00561.x 20723133

[B9] Chugh A. Amundsen E. Eudes F. (2009). Translocation of cell-penetrating peptides and delivery of their cargoes in triticale microspores. Plant Cell Rep. 28, 801–810. 10.1007/s00299-009-0692-4 19288265

[B10] Derossi D. Joliot A. H. Chassaing G. Prochiantz A. (1994). The third helix of the Antennapedia homeodomain translocates through biological membranes. J. Biol. Chem. 269, 10444–10450. 8144628

[B11] Folling L. Olesen A. (2001). Transformation of wheat (*Triticum aestivum* L.) microspore-derived callus and microspores by particle bombardment. Plant Cell Rep. 20, 629–636. 10.1007/s002990100371

[B12] Fonseca S. B. Pereira M. P. Kelley S. O. (2009). Recent advances in the use of cell-penetrating peptides for medical and biological applications. Adv. Drug Deliv. Rev. 61, 953–964. 10.1016/j.addr.2009.06.001 19538995

[B13] Frankel A. D. Pabo C. O. (1988). Cellular uptake of the tat protein from human immunodeficiency virus. Cell 55, 1189–1193. 10.1016/0092-8674(88)90263-2 2849510

[B14] Futaki S. (2002). Arginine-rich peptides: potential for intracellular delivery of macromolecules and the mystery of the translocation mechanisms. Int. J. Pharm. 245, 1–7. 10.1016/S0378-5173(02)00337-X 12270237

[B15] Gaj T. Guo J. Kato Y. Sirk S. J. Barbas C. F. III. (2012). Targeted gene knockout by direct delivery of zinc-finger nuclease proteins. Nat. Methods 9, 805–807. 10.1038/nmeth.2030 22751204 PMC3424280

[B16] Green M. Loewenstein P. M. (1988). Autonomous functional domains of chemically synthesized human immunodeficiency virus tat trans-activator protein. Cell 55, 1179–1188. 10.1016/0092-8674(88)90262-0 2849509

[B17] Hallbrink M. Floren A. Elmquist A. Pooga M. Bartfai T. Langel U. (2001). Cargo delivery kinetics of cell-penetrating peptides. Biochim. Biophys. Acta 1515, 101–109. 10.1016/S0005-2736(01)00398-4 11718666

[B18] Hu T. Kasha K. J. (1999). A cytological study of pretreatments used to improve isolated microspore cultures of wheat (*Triticum aestivum* L.) cv. Chris. Genome 42, 432–441. 10.1139/gen-42-3-432

[B19] Huang Y. Jiang Y. Wang H. Wang J. Shin M. C. Byun Y.. (2013). Curb challenges of the “Trojan Horse” approach: Smart strategies in achieving effective yet safe cell-penetrating peptide-based drug delivery. Adv. Drug Deliv. Rev. 65, 1299–1315. 10.1016/j.addr.2012.11.007 23369828 PMC3657576

[B20] Hul T. Kasha K. J. (1997). Improvement of isolated microspore culture of wheat (*Triticum aestivum* L.) through ovary co-culture. Plant Cell Rep. 16, 520–525. 10.1007/BF01142316 30727571

[B21] Kumlehn J. Serazetdinova L. Hensel G. Becker D. Loerz H. (2006). Genetic transformation of barley (*Hordeum vulgare* L.) via infection of androgenetic pollen cultures with *Agrobacterium tumefaciens*. Plant Biotechnol. J. 4, 251–261. 10.1111/j.1467-7652.2005.00178.x 17177801

[B22] Laurie D. A. Bennett M. D. (1988a). The production of haploid wheat plants from wheat × maize crosses. Theor. Appl. Genet. 76, 393–397. 10.1007/BF00265339 24232203

[B23] Laurie D. A. Bennett M. D. (1988b). Cytological evidence for fertilization in hexaploid wheat × sorghum crosses. Plant Breed. 100, 73–82. 10.1111/j.1439-0523.1988.tb00220.x

[B24] Lindgren M. Langel U. (2011). Classes and prediction of cell-penetrating peptides. Methods Mol. Biol. 683, 3–19. 10.1007/978-1-60761-919-2_1 21053118

[B25] Liu B. R. Liou J. S. Huang Y. W. Aronstam R. S. Lee H. J. (2013). Intracellular delivery of nanoparticles and DNAs by IR9 cell-penetrating peptides. PLoS ONE 8:e64205. 10.1371/journal.pone.0064205 23724035 PMC3665793

[B26] Liu J. Gaj T. Patterson J. T. Sirk S. J. Barbas C. F. III. (2014). Cell-penetrating peptide-mediated delivery of TALEN proteins via bioconjugation for genome engineering. PLoS ONE 9:e85755. 10.1371/journal.pone.0085755 24465685 PMC3896395

[B27] Liu J. Gaj T. Wallen M. C. Barbas C. F. III. (2015). Improved cell-penetrating zinc-finger nuclease proteins for precision genome engineering. Mol. Ther. Nucleic Acids 4, e232. 10.1038/mtna.2015.6 25756962 PMC4354341

[B28] Oehlke J. Scheller A. Wiesner B. Krause E. Beyermann M. Klauschenz E.. (1998). Cellular uptake of an alpha-helical amphipathic model peptide with the potential to deliver polar compounds into the cell interior non-endocytically. Biochim. Biophys. Acta 1414, 127–139. 10.1016/S0005-2736(98)00161-8 9804921

[B29] Pooga M. Hällbrink M. Zorko M. Langel U. (1998). Cell penetration by transportan. FASEB J. 12, 67–77. 9438412 10.1096/fasebj.12.1.67

[B30] Quilichini T. D. Friedmann M. C. Samuels A. L. Douglas C. J. (2010). ATP-binding cassette transporter G26 is required for male fertility and pollen exine formation in arabidopsis. Plant Physiol. 154, 678–690. 10.1104/pp.110.161968 20732973 PMC2949020

[B31] Ramakrishna S. Kwaku Dad A. B. Beloor J. Gopalappa R. Lee S. K. Kim H. (2014). Gene disruption by cell-penetrating peptide-mediated delivery of Cas9 protein and guide RNA. Genome Res. 24, 1020–1027. 10.1101/gr.171264.113 24696462 PMC4032848

[B32] Resch T. Touraev A. (2010). Pollen transformation technologies, in Plant Transformation Technologies, eds Stewart C. N. Touraev A. Citovsky V. Tzfira T. (Oxford, UK: Wiley-Blackwell), 83–91. 10.1002/9780470958988.ch5

[B33] Salter J. Murray B. G. Braggins J. E. (2002). Wettable and unsinkable: the hydrodynamics of saccate pollen grains in relation to the pollination mechanism in the two New Zealand species of Prumnopitys Phil. (Podocarpaceae). Ann. Bot. 89, 133–144. 10.1093/aob/mcf019 12099344 PMC4233786

[B34] Scheller A. Oehlke J. Wiesner B. Dathe M. Krause E. Beyermann M.. (1999). Structural requirements for cellular uptake of α-helical amphipathic peptides. J. Pept. Sci. 5, 185–194. 10323198 10.1002/(SICI)1099-1387(199904)5:4<185::AID-PSC184>3.0.CO;2-9

[B35] Sinha R. Eudes F. (2015). Dimethyl tyrosine conjugated peptide prevents oxidative damage and death of triticale and wheat microspores. Plant Cell Tissue Organ Cult. 122, 227–237. 10.1007/s11240-015-0763-x

[B36] Tang X. Liu Y. He Y. Ma L. Sun M.-x. (2013). Exine dehiscing induces rape microspore polarity, which results in different daughter cell fate and fixes the apical–basal axis of the embryo. J. Exp. Bot. 64, 215–228. 10.1093/jxb/ers327 23162119 PMC3528033

[B37] Touraev A. Indrianto A. Wratschko I. Vicente O. Heberle-Bors E. (1996). fficient microspore embryogenesis in wheat (*Triticum aestivum* L.) induced by starvation at high temperature. Sex. Plant Reprod. 9, 209–215. 10.1007/BF02173100

[B38] Vivès E. Brodin P. Lebleu B. (1997). A Truncated HIV-1 tat protein basic domain rapidly translocates through the plasma membrane and accumulates in the cell nucleus. J. Biol. Chem. 272, 16010–16017. 10.1074/jbc.272.25.16010 9188504

[B39] Voytas D. F. Gao C. (2014). Precision genome engineering and agriculture: opportunities and regulatory challenges. PLoS Biol. 12:e1001877. 10.1371/journal.pbio.1001877 24915127 PMC4051594

[B40] Wang F. Wang Y. Zhang X. Zhang W. Guo S. Jin F. (2014b). Recent progress of cell-penetrating peptides as new carriers for intracellular cargo delivery. J. Control. Release 174, 126–136. 10.1016/j.jconrel.2013.11.020 24291335

[B41] Wang Y. Cheng X. Shan Q. Zhang Y. Liu J. Gao C.. (2014a). Simultaneous editing of three homoeoalleles in hexaploid bread wheat confers heritable resistance to powdery mildew. Nat. Biotechnol. 32, 947–951. 10.1038/nbt.2969 25038773

[B42] Zadoks J. C. Chang T. T. Konzak C. F. (1974). A decimal code for the growth stages of cereals. Weed Res. 14, 415–421. 10.1111/j.1365-3180.1974.tb01084.x

