# Supplementary material for: Intracellular delivery of fluorescent protein into viable wheat microspores using cationic peptides
Source: Front Plant Sci. 2015 Aug 28;6:666. doi: 10.3389/fpls.2015.00666 (PMC4552043; doi:10.3389/fpls.2015.00666)
Supplement: Supplementary file 1 [file DataSheet1.DOCX]

**
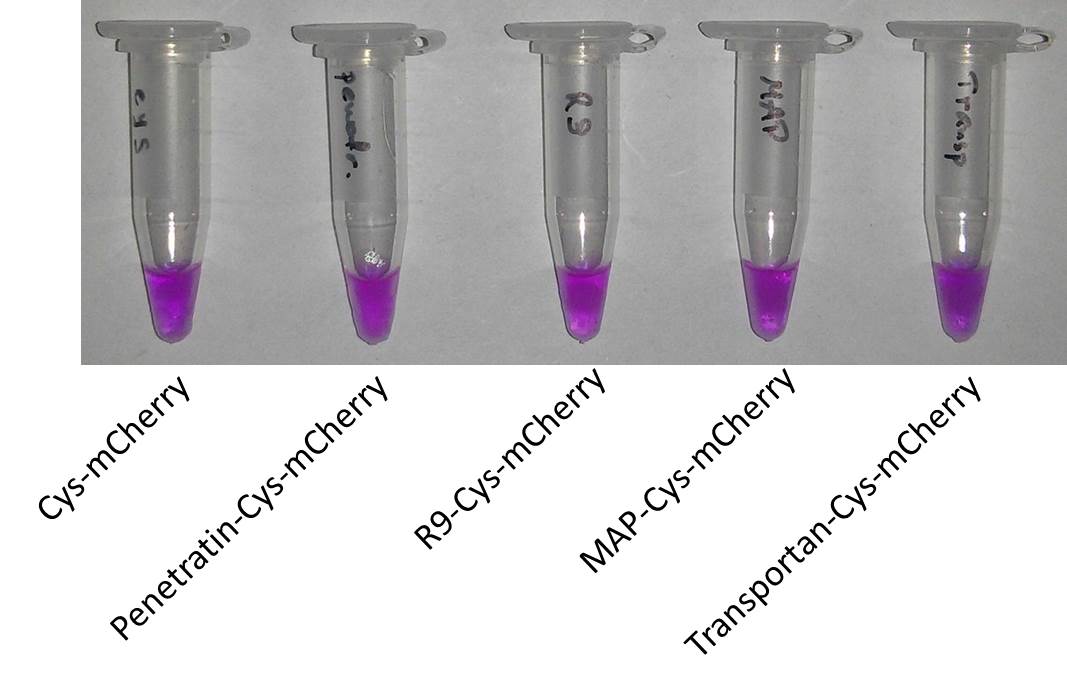
**

**Supplementary figure 1.** Aliquots of corresponding CPP-mCherry proteins demonstrating the intactness of mCherry protein confirmation regardless of the nature of CPP fused

**Supplementary table 1.** PCR primers used in the study

| AB110 | AAACCATGGCATGTATGGCCGTTTCTAAGGGCGAGG | Forward primer for amplification of mCherry CDS, contains TGT codon for Cys and NcoI restriction site. |
| --- | --- | --- |
| AB111 | AAAAAGCTTTCAGTGATGGTGGTGGTGATGCTTGTACAGCTCGTCCATTC | Reverse primer for amplification of mCherry CDS, has HIS-tag sequence and HindIII restriction site |
| AB112 | AAACCATGGCACGTCGTCGTCGTCGTCGTCGTCGTCGTTGTATGGCCGTTTCTAAGGGCGA | Forward primer for amplification of mCherry CDS, has R9 sequence, TGT codon for Cys and NcoI restriction site. |
| AB113 | AAACCATGGCACGTCAGATTAAAATTTGGTTTCAGAACCGTCGTATGAAATGGAAAAAATGTATGGCCGTTTCTAAGGGCGA | Forward primer for amplification of mCherry CDS, has Penetratin sequence, TGT codon for Cys and NcoI restriction site. |
| AB127 | AAACCATGGCAGGCTGGACCCTGAACAGCGCGGGCTATCTGCTGGGCAAAATTAACCTGAAAGCGCTGGCGGCGCTGGCGAAAAAAATTCTGTGTATGGCCGTTTCTAAGGGCGA | Forward primer for amplification of mCherry CDS, has Transportan sequence, TGT codon for Cys and NcoI restriction site. |
| AB115 | AAACCATGGCAAAACTGGCGCTGAAACTGGCGCTGAAAGCGCTGAAAGCGGCGCTGAAACTGGCGTGTATGGCCGTTTCTAAGGGCGA | Forward primer for amplification of mCherry CDS, has MAP sequence, TGT codon for Cys and NcoI restriction site. |
